# Frequency of Cancer Genes on the Chicken Z Chromosome and Its Human Homologues: Implications for Sex Chromosome Evolution

**DOI:** 10.1155/2007/43070

**Published:** 2007-01-08

**Authors:** Rami Stiglec, Matthias Kohn, James Fong, Tariq Ezaz, Horst Hameister, Jennifer A. Marshall Graves

**Affiliations:** ^1^Comparative Genomics Group, Research School of Biological Sciences, The Australian National University, Canberra ACT 0200, Australia; ^2^Department of Human Genetics, University of Ulm, 89070 Ulm, Germany

## Abstract

It has been suggested that there are special evolutionary forces that act on sex chromosomes. Hemizygosity of the X chromosome in male mammals has led to selection for male-advantage genes, and against genes posing extreme risks of tumor development. A similar bias against cancer genes should also apply to the Z chromosome that is present as a single copy in female birds. Using comparative database analysis, we found that there was no significant underrepresentation of cancer genes on the chicken Z, nor on the Z-orthologous regions of human chromosomes 5 and 9. This result does not support the hypothesis that genes involved in cancer are selected against on the sex chromosomes.

## 1. INTRODUCTION

In humans, and most other mammals, sex is determined by an XY male: XX female system in which the *SRY* gene on the Y chromosome determines testis development [1]. The Y chromosome represents a degraded relic of the X and has been left with only 45 genes of the 1000 or so it originally had, which are now represented solely on the X [2]. In female placental mammals, X inactivation randomly silences one X chromosome, thus maintaining a balanced dosage of X-linked gene products between the sexes.

For an autosomal gene, the missing or inactive products of recessive alleles are complemented by the normal product of the wild-type allele in heterozygotes, producing a wild-type, or intermediate, phenotype. However, hemizygosity for the X chromosome poses a quandary for males in that deleterious recessive mutations of X-borne genes will have an immediate lethal or harmful effect on phenotype. The absence of a second allele to compensate for the recessive mutant allele means that the frequency of deleterious sex linked recessive conditions (such as colour blindness, haemophilia, and many forms of mental retardation) is far higher in males than females. The same would be true for an X-borne gene in females due to X inactivation, except that heterozygotes are mosaics for mutant and wild-type tissue, so that phenotype is less severe.

This bias poses a particular problem for genes within which somatic mutation would be lethal, including genes that control somatic growth. It was proposed that genes whose absence cause unregulated growth have been selected against on the human X chromosome, leaving the X depauperate in these “cancer genes” [3].

Genes implicated in cancer can be defined as those that control cell growth, and whose constitutional or somatic mutations cause tumors. There are two classes of such genes: oncogenes, which promote cell growth; and tumor suppressor (TS) genes, which inhibit excess growth. Cancer is initiated after loss-of-function mutations in both alleles of autosomal TS genes. Loss-of-function mutations in such genes may therefore act as dominant cancer susceptibility mutations (e.g., retinoblastoma [4]); since in the presence of a constitutional mutation of one allele, mutation of the wild-type allele in somatic tissue during life produces an early onset of tumor. Gain-of-function mutations of oncogenes also result in cancer because of disruption of the stringent transcriptional control necessary for their cell-autonomous activity [5]. XY males would therefore be particularly susceptible to cancer caused by somatic mutations of X-linked cancer genes.

A paucity of potentially cancer-causing genes from the X chromosome has been suggested to be the result of selection against such genes [3, 6]. There is a striking absence of potent growth-related and tumor suppressor genes on the human X chromosome [3, 6]. Potent oncogenes are also absent from the X chromosome, and there are few examples of tumor-specific activating mutations on the X, such as translocations and inversions, that could involve oncogene activation [3]. Only a few relatively benign TS genes (such as the prostate cancer susceptibly loci *AR* and *HPCX*) are found on the X chromosome [7].

More recently, many families of genes expressed in sperm and reexpressed in tumors (testis-cancer antigens) have been discovered on the human X chromosome (listed in the NCBI database), most of which are members of large gene families (e.g., CT45, SSX, SPANX, and MAGE) resulting from gene amplifications [8]. These amplified genes are mammalian or primate specific [8, 9] and many of them have important spermatogenesis or sex-specific functions [10]. Cancer genes with paralogues that could complement their function would not pose the same risks as single copy genes on the X. When these amplified genes are discounted, there seems to have been a selection against genes on the X chromosome with essential cellular functions; mutations in which would cause tumors.

Is the bias against cancer genes a general characteristic of sex chromosomes? This question may be answered by studying birds, in which the female rather than the male is the heterogametic sex. Males have two copies of the large and gene rich Z chromosome, whereas females have a single copy of the Z, plus the small and heterochromatic W chromosome. Although there appears to be some degree of Z-dosage compensation in birds [11], RNA FISH shows that alleles of both Z chromosomes are expressed, so that male ZZ birds are true heterozygotes [12]. We would therefore expect that the hemizygous ZW female bird is as vulnerable to mutations in Z-linked cancer genes as is the XY male mammal. The chicken Z is therefore predicted to bear disproportionately fewer cancer genes.

According to the generally accepted view, mammalian X/Y and avian Z/W sex chromosomes evolved independently from two different pairs of ancestral autosomes in a common ancestor with no sex chromosomes [13–15]. Comparative gene mapping between human and chicken (*Gallus gallus*) supports this hypothesis. Mammalian X chromosome genes localise to autosomes in chicken (mostly GGA 1, 4, and 12 [16, 17]), whereas chicken Z genes are largely found in segments of two human (*Homo sapiens*) autosomes (HSA) 5 and 9, and a smaller segment on 18 [18]. The hypothesis that mammal XX/XY and bird ZZ/ZW systems evolved independently from different autosomal pairs predicts that the chicken and human autosomal regions orthologous to the sex chromosomes (X and Z) would not have been subject to purifying selection against cancer genes. However, the alternative hypothesis that ancestral mammals shared the ZW system with birds, supported by the extraordinary complex sex chromosome system of the platypus [19], predicts that the Z-orthologous regions of HSA 5 and 9 would still bear a bias against cancer genes.

It should therefore be possible to locate and characterise cancer genes on sex chromosomes and their autosomal orthologues, and determine if any were lost from the sex chromosomes of one lineage or the other. Here we test the hypothesis that, like the mammal X, the bird Z underwent sex-chromosome-specific cleansing of cancer genes to protect the heterogametic sex from tumor-causing mutations, and predict that the chicken Z chromosome is depleted of cancer genes, as is the human X.

Since we have little direct knowledge of avian cancer genes, most information is available for the chicken Z via its human orthologues. We therefore searched for oncogenes and TS genes on the regions on human chromosomes 5 and 9 orthologous to the chicken Z chromosome, and then searched for chicken orthologous of these genes. We used the non-Z-homologous regions of the same human chromosomes as controls. We demonstrated that the frequency of cancer genes is the same on the chicken Z and autosomes, suggesting that the chicken Z chromosome has not undergone a purification of cancer genes to protect hemizygotic females. Nor did we find significant difference between the frequency of cancer genes versus noncancer genes in the Z-homologous and chicken autosomal-homologous regions of human chromosomes 5 and 9, consistent with the hypothesis that the mammal XY and bird ZW sex chromosome systems evolved independently.

## 2. METHODS

Using the web-based NCBI and UCSC human and chicken databases we looked at 1, 876 HSA 5 and 9 protein coding genes (981 and 895, resp.) and constructed a detailed chicken-human comparative map of these chromosomes. The location of chicken orthologues of human genes in the chicken genome was determined via the UCSC Chicken Genome Browser Gateway on the latest assembly of the chicken genome (May 2006, galGal3).

An index of human cancer genes, with corresponding chromosomal locations, was compiled from the NCBI database (key search words were “human cancer,” “human tumor suppressor,” and “human oncogene”). This index was used to extract a list of 175 cancer genes on HSA 5 (Table 1) and HSA 9 (Table 2), within and outside the regions of orthology with the chicken Z. Each of these cancer genes was then used to screen the chicken databases and the positions of these orthologues on chicken chromosome were established (Table 3).

## 3. RESULTS

There have been no comprehensive comparative analyses of the locations of cancer genes in the chicken genome. Our strategy was therefore to use a comparative bioinformatics approach to identify the regions of HSA 5 and 9 homologous to the chicken Z, and outside the regions of homology, to identify cancer genes within these human chromosome regions, then to ascertain how many of these were retained on the chicken Z versus chicken autosomes.

There was a total of 175 cancer genes on HSA 5 and 9. Of these, 82 lay within the Z-homologous regions, and 93 lay outside these regions. The highest concentration of cancer genes in both HSA 5 and 9 was located on the terminal regions of their long arms.

Of the 175 human cancer genes, 164 had clear orthologues in the chicken genome; seven were absent from the chicken genome. Of the seven apparently missing from the chicken genome, two (SPINK4 and CCL21) lay in Z-homologous regions of HSA 5 and HSA 9, and nine lay in non-Z-homologous regions of these chromosome. tBLASTx searches for SPINK4 and CCL21 return moderate-level hits on the chicken genome (accession numbers BX934389 and CR522995, resp.) both of which are Z-link sequences.

This suggests that although these genes might be Z-linked in chickens, their sequences have drastically diverged at the nucleotide level; as such, they have not been included in our analysis. Of the 164 chicken orthologues, 72 localised to the chicken Z chromosome (Table 3). There were only four genes in the Z-homologous region that mapped to chicken autosomes.

Of the remainder of the human cancer genes with detectable orthologues in the chicken genome, 92 localised to chicken autosomes and four had not yet been localised to a specific chromosome (although all four were found in Z-homologous regions and could be Z genes; the uncertainty of their location, however, meant that they were not included in our analysis) (Table 3). Nearly all the non-Z-homologous HSA 5 genes mapped to GGA 13 and most non-Z-homologous HSA 9 genes to GGA 17, with six on GGA 2 and a few singletons (possibly mis-mapped or misidentified). GGA 13, which shows partial colinearity with HSA 5, has 44 cancer genes; and GGA 17, which, with the exception of a few inversions, demonstrates colinearity with HSA 9, has 32 cancer genes.

We then compared the frequencies of human cancer genes among total genes in the Z-homologous regions of HSA 5 and 9 (9.3%) and the autosome-homologous regions of HSA 5 and 9 (10.0%), which are not significantly different by a chi-squared test on frequencies (Table 4). Then we compared the frequencies of cancer genes on the chicken Z (13.7%) with the frequencies of the autosomal regions on the rest of HSA 5 and HSA 9 (13.9%), which is also nonsignificant.

## 4. DISCUSSION

An underrepresentation of tumour suppressor genes and oncogenes on the human X chromosome [3, 6] has been explained by the hypothesis that such genes are selected against because of their propensity for somatic mutation to cause cancer. Hameister and Adolph's [3] original claim that the human X chromosome is depleted of cancer genes has been challenged by new analyses of human genome data, which show that the human X contains many primate-specific families of cancer genes such as the synovial sarcoma X (SSX) breakpoint family; the cancer/testis antigen families: CT45, CTAG, SPANX, plus the GAGE cancer/testis antigen subfamilies: XAGE, PAGE, and MAGE, which appear to be amplified within large palindromes. These cancer/testis antigen genes play an essential role in normal testis development and function and may be just upregulated in tumour tissues rather than involved in tumourigenesis. If these duplicated genes are discounted, the human X does indeed appear to be depleted in cancer genes.

This hypothesis implies that once an X chromosomal region stopped undergoing recombination with the proto-Y chromosome, there was strong selection for loss of cancer genes on the X (deleted or translocated to an autosome), or loss of their cancer-causing function. Only physical loss could be detected by our comparative bioinformatics strategy.

Our study provides no evidence that selection against cancer genes has occurred on the chicken Z chromosome. We observed that the frequency of cancer gene orthologues on the chicken Z is not significantly different from the frequency of cancer gene orthologues on the chicken autosome regions that share HSA 5 and HSA 9 with the Z. Nor is the frequency of cancer genes within the Z-homologous and Z-nonhomologous regions of HSA 5 and 9 different.

This result does not support the hypothesis that cancer genes on the Z are selected against because of their hemizygotic presence in female chickens. Thus paucity of cancer genes is not a universal characteristic of sex chromosomes.

A major source of uncertainly in this study is the definition of “cancer genes” in humans and chickens, and whether the chicken orthologues of human cancer genes are also involved in cancer. Many of the human cancer genes on HSA 5 and 9 are involved in breast cancer, so may have a cancer-causing potential only in mammals. The exact role of many human (let alone chicken) growth-regulating genes in disease is unknown. Although some genes in the human cancer gene database we used were identified as oncogenes or tumour suppressors (Tables 1 and 2), it is uncertain whether these genes actually initiate tumour development or they are merely upregulated in cancer tissues by other upstream genes. Many genes (particularly the amplified primate-specific genes) were labeled as testis-cancer antigens on the basis of their re-expression in some cancers. Because there are few direct data concerning avian cancer genes, we had to make the working assumption that chicken homologues of human cancer genes are also involved in avian cancer. Although we cannot be certain that human cancer genes are involved with cancer in chickens, all human oncogenes and TS genes have essential cell-cycle functions, so conserved orthologues are expected to possess similar roles.

There is an apparent loss of cancer genes from the human X, but not the chicken Z, which suggests that the cancer gene contents of the X and Z are under different selection pressures. This could be a function of the different life spans between chickens and humans. Hunter and Cozma [20] demonstrated that cancer latency and life span are linked—cancer acts as a somatic recessive in long-lived species such as humans, but a dominant in short-lived species such as mice, which reproduce before they succumb to a tumour.

We observed, also, that the regions of human chromosomes 5 and 9 that are orthologous to chicken Z were not significantly depleted of cancer genes, implying that these regions do not bear the legacy of once having been sex chromosomes, as suggested by Grützner et al. [19].

Our analysis reveals that cancer genes occur at the same frequency on the chicken Z chromosome as on chicken autosomes. Thus, we have established that hemizygotic selection pressures on cancer genes are not a universal characteristic of heterogametic sex chromosomes. This challenges the assumption that both XX/XY and ZZ/ZW systems are subject to similar sex-chromosome-specific evolutionary selection pressures, and urges caution in interpretation of observations on biased gene content of sex chromosomes.

## Figures and Tables

**Table 1:** List of 89 cancer genes from human chromosome 5 and their locations in the chicken genome.

Gene Symbol^1^	Accession number	Type of cancer gene	Chromosome location in human	Chromosome location in chicken

AHRR	NM_020731	—	5p15.3	2
TERT	NM_198253	Oncogene	5p15.33	2
SRD5A1	NM_001047	—	5p15	2
AMACR	NM_014324	—	5p13.2-q11.1	Z
PRLR	NM_000949	—	5p14-p13	Z
SKP2	NM_005983	TS^2^	5p13	Z
GDNF	NM_000514	—	5p13.1-p12	Z
DAB2	NM_001343	TS	5p13	Z
GHR	NM_000163	—	5p13-p12	Z
ITGA1	NM_181501	—	5q11.2	Z
ITGA2	NM_002203	—	5q23-q31	Z
GZMA	NM_006144	TS	5q11-q12	Z
PPAP2A	NM_176895	—	5q11	Z
RAB3C	NM_138453	Oncogene	5q13	Z
MAP3K1	XM_424734	—	5q11.2	Z
SDCCAG10	NM_005869	—	5q12.3	Z
PIK3R1	NM_181523	—	5q13.1	Z
CCNB1	NM_031966	—	5q12	10
RAD17	NM_133338	—	5q13	Z
OCLN	NM_002538	—	5q13.1	Z
ENC1	NM_003633	TS	5q12-q13.3	Z
F2R	NM_001992	—	5q13	Z
F2RL1	NM_005242	—	5q13	Z
MSH3	NM_002439	—	5q11-q12	Z
SSBP2	NM_012446	TS	5q14.1	Z
XRCC4	NM_022406	—	5q13-q14	Z
CSPG2	NM_004385	—	5q14.3	Z
GLRX	NM_205160	—	5q14	Z
ELL2	NM_012081	TS	5q15	Z
PCSK1	NM_000439	—	5q15-q21	Z
FER	NM_005246	—	5q21	Z
CAMK4	NM_001744	—	5q21.3	Z
APC	NM_000038	TS	5q21-q22	Z
MCC	NM_002387	TS	5q21-q22	Z
TRIM36	NM_018700	TS	5q22.3	Z
PGGT1B	NM_005023	—	5q22.3	Z
CCDC112	NM_001040440	—	5q22.3	Z
TNFAIP8	NM_014350	—	5q23.1	Z
LOX	NM_002317	TS	5q23.2	Z
HINT1	NM_005340	TS	5q31.2	Z
GMCSF	NM_001007078	—	5q31.1	13
IRF1	NM_002198	TS	5q31.1	13
IL4	NM_001007079	—	5q31.1	13
AFF4	NM_014423	—	5q31	13
HSPA4	NM_002154	—	5q31.1-q31.2	13
TCF7	NM_003202	TS	5q31.1	13
TGFBI	NM_000358	TS	5q31	13
KIF20A	NM_005733	TS	5q31	13
CDC23	NM_004661	TS	5q31	13
JMJD1B	NM_016604	TS	5q31	13
EGR1	NM_001964	TS	5q31.1	13
HSPA9B	NM_004134	TS	5q31.1	13
CTNNA1	NM_001903	—	5q31	13
PURA	NM_005859	TS	5q31	13
SRA1	NM_001035235	—	5q31.3	13
HDAC3	NM_003883	TS	5q31	13
RNF14	NM_004290	—	5q23.3-q31.1	13
FGF1	NM_000800	—	5q31	13
NR3C1	NM_001018077	TS	5q31.3	13
PPP2R2B	NM_004576	TS	5q31-5q32	13
SPINK1	NM_003122	—	5q32	4
SPINK5	NM_006846	—	5q32	13
SPINK5L2	NM_001001325	—	5	—
SPINK5L3	NM_001040129	—	5q32	—
ECG2	NM_032566	—	5q32	—
CSNK1A1	NM_001025105	—	5q32	13
CSF1R	NM_005211	Oncogene	5q33-q35	13
PDGFRB	NM_002609	—	5q31-q32	13
CDX1	NM_001804	—	5q31-q33	13
FAT2	NM_001447	TS	5q32-q33	13
SPARC	NM_003118	—	5q31.3-q32	13
ATOX1	NM_004045	—	5q32	13
IL12B	NM_002187	—	5q31.1-q33.1	13
PTTG1	NM_004219	—	5q35.1	13
CCNG1	NM_004060	TS	5q32-q34	13
HMMR	NM_012484	—	5q33.2-qter	13
TLX3	NM_021025	Oncogene	5q35.1	13
NPM1	NM_002520	TS	5q35	13
FGF18	NM_003862	Oncogene	5q34	13
DUSP1	NM_004417	—	5q34	13
UNC5A	NM_133369	TS	5q35.2	13
FGFR4	XM_414474	—	5q35.1-qter	13
RAB24	NM_001031677	Oncogene	5q35.3	13
NOLA2	NM_017838	—	5q35.3	13
SQSTM1	NM_003900	—	5q35	13
MAPK9	NM_002752	TS	5q35	13
FLT4	NM_182925	—	5q34-q35	13
SCGB3A1	NM_052863	TS	5q35-qter	13
GNB2L1	NM_006098	Oncogene	5q35.3	16

^1^Human gene symbol.

^2^TS: tumor suppressor gene.

**Table 2:** List of 86 cancer genes from human chromosome 9 and their locations in the chicken genome.

Gene Symbol^1^	Accession number	Type of cancer gene	Chromosome location in human	Chromosome location in chicken

SMARCA2	NM_003070	TS^2^	9p22.3	Z
ANKRD15	NM_015158	TS	9p24	Z
JAK2	NM_004972	—	9p24	Z
CD274	NM_014143	—	9p24	Z
PDCD1LG2	NM_025239	—	9p24.2	Z
MLLT3	NM_004529	—	9p22	Z
IFNB1	NM_002176	TS	9p21	Unknown^3^
IFNA17	NM_021268	—	9p22	Unknown
MTAP	NM_002451	TS	9p21	Z
CDKN2A	NM_204433	TS	9p21	Z
CDKN2B	NM_004936	TS	9p21	8
TUSC1	NM_001004125	TS	9p21.2	Z
TOPORS	NM_005802	TS	9p21	Z
B4GALT1	NM_001497	—	9p13	Z
SPINK4	NM_014471	—	9p13.3	—
BAG1	NM_004323	—	9p12	2
UBAP1	NM_016525	TS	9p22-p21	Z
NUDT2	NM_001161	TS	9p13	Z
GALT	NM_000155	—	9p13	Z
IL11RA	NM_004512	—	9p13	Z
CCL21	NM_002989	—	9p13	—
FANCG	NM_204378	—	9p13	Z
CA9	NM_001216	TS	9p13-p12	Z
TLN1	NM_006289	TS	9p13	Z
RECK	NM_021111	—	9p13-p12	2
PAX5	NM_016734	Oncogene	9p13	Z
IGFBPL1	NM_001007563	TS	9p13.1	4
ALDHA1	NM_000689	—	9q21.13	Z
ANXA1	NM_000700	TS	9q12-q21.2	Z
GCNT1	NM_001490	—	9q13	Z
UBQLN1	NM_013438	Oncogene	9q21.2-q21.3	Z
NTRK2	NM_006180	Oncogene	9q22.1	Z
GAS1	NM_002048	TS	9q21.3-q22	Z
DAPK1	NM_004938	Oncogene	9q34.1	Z
CTSL	NM_001912	—	9q21-q22	Z
SHC3	NM_016848	—	9q22.1-q22.2	Z
GADD45G	NM_006705	TS	9q22.1-q22.2	Unknown
SYK	NM_003177	TS	9q22	Z
WNK2	NM_006648	—	9q22.3	12
FANCC	NM_000136	TS	9q22.3	Z
PTCH1	NM_000264	TS	9q22.3	Z
CDC14B	NM_033331	TS	9q22.33	Z
XPA	NM_204853	—	9q22.3	Z
ANP32B	NM_006401	TS	9q22.32	28
GALNT12	NM_024642	—	9q22.33	2
TGFBR1	NM_004612	—	9q22	2
NR4A3	NM_006981	—	9q22	2
TMEFF1	NM_003692	TS	9q31	2
KLF4	NM_004235	TS	9q31	Unknown
TXN	NM_003329	—	9q31	Z
EDG2	NM_001401	—	9q31.3	Z
UGCG	NM_003358	—	9q31	Z
AMBP	NM_001633	—	9q32-q33	17
TNFSF15	NM_005118	—	9q32	17
TNC	NM_002160	—	9q33	17
DEC1	NM_017418	TS	9q32	—
TRIM32	NM_012210	Oncogene	9q31-q34.1	17
TLR4	NM_138554	—	9q32-q33	17
DBC1	NM_014618	TS	9q32-q33	17
TRAF1	NM_005658	—	9q33-q34	17
RAB14	NM_016322	Oncogene	9q32-q34.11	17
GSN	NM_000177	—	9q33	17
DAB2IP	NM_032552	TS	9q33.1-q33.3	17
PTGS1	NM_000962	—	9q32-q33.3	17
NR5A1	NM_004959	TS	9q33	17
FPGS	NM_004957	—	9q34.1	17
ENG	NM_000118	—	9q33-q34.1	17
LCN2	NM_005564	Oncogene	9q34	—
SET	NM_003011	TS	9q34	17
PKN3	NM_013355	TS	9q34.11	17
PTGES	NM_004878	TS	9q34.3	17
ABL1	NM_005157	Oncogene	9q34.1	17
NUP214	NM_005085	Oncogene	9q34.1	17
RAPGEF1	NM_198679	Oncogene	9q34.3	17
TSC1	NM_000368	TS	9q34	17
RALGDS	NM_001042368	TS	9q34.3	17
RPL7A	NM_001004379	Oncogene	9q34	17
SURF1	NM_003172	Oncogene	9q34	17
ADAMTS13	NM_139025	–	9q34	17
VAV2	NM_003371	Oncogene	9q34.1	17
RXRA	NM_002957	—	9q34.3	17
SDCCAG3	NM_001039707	—	9q34.3	17
NOTCH1	NM_017617	TS	9q34.3	17
AGPAT2	NM_006412	—	9q34.3	17
COBRA1	NM_015456	—	9p34	17
NOXA1	NM_006647	—	9q34.3	17

^1^Human gene symbol.

^2^TS: tumor suppressor gene.

^3^Unknown: the nucleotide sequence of the gene is annotated in the chicken genome assembly but its chromosomal location is not yet known.

**Table 3:** Summary of the localisations of cancer genes from human chromosomes 5 and 9 in the chicken genome.

Chicken chromosomes (GGA)	Human chromosomes (HSA)		Total

	5	9	

Z	36	36	72
2	3	6	9
4	1	1	2
8	—	1	1
10	1	—	1
12	—	1	1
13	44	—	44
16	1	—	1
17	—	32	32
28	—	1	1

Autosomal total	50	42	92

Unknown chromosome	0	4	4
Mammal-specific	3	4	7

Combined total	89	86	175

**Table 4:** Frequencies of cancer genes versus total genes in regions of human chromosomes 5 and 9 that are homologous to the chicken Z chromosome and to chicken autosomes. Human genes refer to all genes within these regions regardless of their homology, or lack thereof, to chicken genes. Chicken genes refer only to genes within these regions that have homolouges in the chicken genome. Chi-square analysis of these frequencies demonstrates that the differences between these regions are not significant.

Genes	Z-homologous regions	Non-Z homologous regions	Chi-square value (1 degree of freedom)

Human genes	78/841 (9.28%)	91/908 (10.02%)	0.23020, *p* ≤ 1
Chicken genes	72/526 (13.69%)	86/621 (13.85%)	0.00468, *p* ≤ 1
